# Adenosine triphosphate stress dual-source computed tomography to identify myocardial ischemia: comparison with invasive coronary angiography

**DOI:** 10.1186/2193-1801-3-75

**Published:** 2014-02-07

**Authors:** Teruhito Kido, Kouki Watanabe, Hideyuki Saeki, Susumu Shigemi, Takeshi Matsuda, Masaya Yamamoto, Akira Kurata, Rene Epunza Kanza, Toshihide Itoh, Teruhito Mochizuki

**Affiliations:** Department of Radiology, Ehime University, Toon, Japan; Department of Cardiology, Saiseikai Matsuyama Hospital, Matsuyama, Japan; Department of Radiology, Saiseikai Matsuyama Hospital, Matsuyama, Japan; Department of Radiology, Sherbrooke University, Quebec, Canada; Research and Collaboration, Siemens Japan, Tokyo, Japan

**Keywords:** Dual Energy CT, Ischemia, Perfusion CT, Myocardium

## Abstract

**Purpose:**

The purpose of this study was to investigate the utility incremental diagnostic value of combined assessment with coronary CT angiography (CCTA) and myocardial CT perfusion imaging (CTP) using dual-energy technology with an Adenosine Triphosphate (ATP) load technique.

**Materials and methods:**

Twenty-one patients underwent ATP-provocation dual-energy CT and CAG. We compared the diagnostic accuracy with CAG, for ischemic region due coronary stenosis by CCTA alone and CCTA combined with CTP (Combined CCTA/CTP).

**Results:**

All of 21 patients CTP images could be evaluated, however 8 CCTA images could not be evaluated by calcification and motion artifact, so assessability was 61.9% (13/21) for CCTA alone, and 100% for Combined CCTA/CTP. With CAG results as a comparison, the sensitivity, specificity, positive predictive value, and negative predictive value were, respectively, 83.3% (20/24), 74.4% (29/39), 66.7% (20/30), and 87.8% (29/33) for CCTA alone, and 66.7% (16/24), 92.3% (36/39), 84.2% (16/19), and 81.8% (36/44) for combined CCTA/CTP. The diagnostic accuracy of the two methods were 77.8% (49/63) and 82.5% (52/63).

**Conclusion:**

Dual-energy CT may be a useful modality for perfusion assessment and correlated well with the severity of stenosis on CAG. This technique may even be of use in cases of severe calcification in the coronary artery wall.

## Introduction

Remarkable advancements in electrocardiography (ECG)-gated multi-slice computed tomography (MSCT), coronary CT angiography (CCTA) has been rapidly spread in clinical practice (Achenbach et al. 1998, 2000, 2001; Nieman et al. 2001; Funabashi et al. 2000). Some studies suggest that noninvasive assessment of coronary artery stenosis and atherosclerotic plaque is useful in assessment of coronary artery disease (CAD) (Leschka et al. 2005; Raff et al. 2005; White et al. 1984; Kern et al. 2006; Tobis et al. 2007; Haraikawa et al. 2006).

Not only coronary artery stenosis, but also an evaluation of myocardial perfusion is essential for diagnosis of myocardial ischemia. Recent advanced studies have investigated the utility of cardiac MSCT to assess myocardial CT perfusion (CTP) using pharmacological stress technique (Kurata et al. 2005; George et al. 2009; Kido et al. 2008; Shikata et al. 2010).

Dual-source CT (DSCT), equipped with two X-ray tubes and two detector arrays mounted in the same gantry, has higher temporal resolution and has been applied to cardiac imaging. Moreover, DSCT has another advantage of dual-energy imaging for tissue differentiation (Johnson et al. 2007; Flohr et al. 2006; Ruzsics et al. 2008; Nagao et al. 2011; Blankstein et al. 2009); with different X-ray spectra, different constructions show different absorption characteristics. Using this technique, Ko et al. reported the diagnostic performance of combined assessment with CCTA and stress dual-energy CTP using double scanning protocol for detection of significant coronary stenosis (Ko et al. 2012). Therefore, this study investigated the utility of single data acquisition of adenosine triphosphate (ATP) stress dual-energy cardiac CT to assess CCTA and CTP in comparison with coronary angiography (CAG) as reference.

## Materials and methods

### Patients

The study protocol was approved by the hospital ethics committee, and informed consent was obtained from all patients. From March 2009 to January 2011, taking the entry and exclusion criteria into account, 21 patients (14 men, 7 women; age range, 59–88 years; mean age, 69.5 years) underwent ATP-provocation contrast-enhanced dual-energy CT, coronary CT angiography, and conventional CAG. The entry criteria were: (i) de novo effort or rest angina (documented ST-T change on ECG, or relieved by administration of nitroglycerin); (ii) no history of coronary artery bypass grafting (CABG); and (iii) asymptomatic patients with multiple coronary risk factors or equivocal or abnormal findings on tredmill test or stress myocardial perfusion single-photon emission computed tomography (SPECT). The exclusion criteria included: (i) a history of myocardial infarction (MI); (ii) unstable angina (onset of angina within the past month; severe or worsening clinical symptoms); (iii) greater than first degree atrio-ventricular block; (iv) renal insufficiency (serum creatinine > 1.5 mg/dl); (v) pregnancy, hyperthyroidism or a known allergic reaction to contrast media; (vi) severe LV dysfunction (LV ejection fraction < 20%); (vii) known history of bronchial asthma, and (viii) New York Heart Association class IV congestive heart failure. Twenty-one cases, in which coronary CT angiography documented abnormal findings, also underwent CAG. The ATP-load dual-energy CT and CAG were performed at an average interval of 18 days. Coronary risk factors among patients were as follows: hypertension (n = 15), diabetes mellitus (n = 7), dyslipidemia (n = 8), and cigarette smoking (n = 7). There were no significant differences between men and women in terms of age, clinical symptoms or coronary risk factors.

### ATP-load cardiac CT protocol

**Figure 1:**
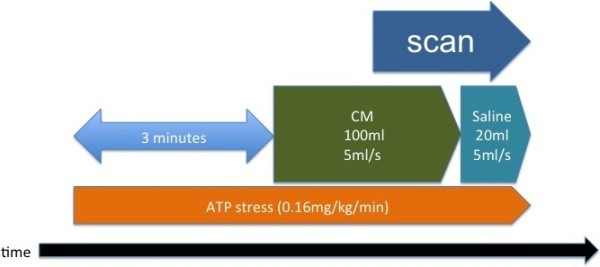
**ATP load cardiac CT protocol.** ATP was infused over 3 minutes at a rate of 0.16 mg/kg/min. 100 ml of contrast medium was injected at a rate of 5 ml/s and followed by 20 ml saline. Scan delay was detected with bolus tracking method at descending aorta, and we set up the delay time for five seconds longer than usually scan to fill the myocardium with contrast medium.

The ATP-load cardiac CT protocol is shown in Figure 1. Patients were scanned in a fasting state in the supine position. Patients were instructed to refrain from caffeine (coffee) intake beginning the evening before the test. ATP (ADETPHOS-L, Kowa, Tokyo, Japan) was infused over 3 min at a rate of 0.16 mg/kg/min using an infusion pump system (Teru-Fusion, Syringe pump. TE-3320C, Terumo, Tokyo, Japan) through a peripheral venous cannula in the cubital vein on the side opposite of that used for the contrast medium infusion. Throughout the infusion, clinical status, heart rate, blood pressure, and ECG were monitored by a cardiologist. Three minutes after the ATP infusion, 100 ml of non-ionic contrast medium (Iopamidol, 370 mg/ml Iopamiron, Bayer Yakuhin Ltd, Osaka, Japan) was injected at a rate of 5 ml/s through a 20-G intravenous antecubital cannula, followed by 20 ml of saline, using a dual-syringe injector (Stellant, Medrad, Indianola, PA, USA). Contrast medium enhancement in the descending aorta was monitored, and after the enhancement reached the descending aorta (threshold of CT value was 150HU), dual-energy scanning was performed during breath-holding. Since peak myocardial enhancement occurs at a time point later than that of peak coronary enhancement, the delay time was set to be 8 s after the enhancement reached the aorta. Immediately after the stress CT, the ATP infusion was discontinued. A dual-source CT (DSCT, SOMATOM Definition and SOMATOM Definition Flash, Siemens Healthcare, Forchheim, Germany) equipped with two pairs of generation-detectors was used. Nine patients underwent dual-energy CT with Definition in 2009, and 12 patients underwent dual-energy CT with Definition Flash from 2010 to 2011. Scan parameters were: 330 ms gantry rotation and 64 × 0.6 mm collimation for Definition; and 275 ms gantry rotation and 128 × 0.6 mm collimation for Definition Flash. Pitches were 0.2 (heart rate: < 60 beats/min), 0.25 (61–70 beats/min), 0.3 (71–80 beats/min), and 0.35 (> 81 beats/min) with Definition; and 0.22 (heart rate: < 60 beats/min), 0.25 (61–70 beats/min), 0.29 (71–80 beats/min), and 0.32 (> 81 beats/min) with Definition Flash. When using Definition, one tube of the dual-source system was operated with 90 mAs at 140 kV, and the other tube was operated with 180 mAs at 100 kV. When using Definition Flash, one tube of the dual-source system was operated with 155 mAs at 140 kV with a tin (Sn) filter for optimization of its X-ray spectrum and the other tube was operated with 185 mAs at 100 kV. Data were acquired in the cranio-caudal direction with simultaneous recording of the patient’s ECG signal to enable retrospective registration of the reconstructed images to the desired cardiac phase. The anatomical range extended from the level of the bronchial bifurcation to just below the dome of the diaphragm. A single oral dose of 25–50 mg atenolol (AstraZeneca Pharmaceuticals, London, UK) was administered 4 h before cardiac CT scanning if the patient’s heart rate exceeded 60 beats/min at that time. No additional medication was given if the heart rate did not decrease sufficiently following this dose. ATP stress scanning was performed in 21 patients; a rest scan was not performed for these patients in order to reduce the overall radiation dose.

### Analysis of ATP stress dual-energy CT

From the single dual-energy CT datasets, three different image reconstructions were performed; the merged 120 kV dataset for coronary CTA, and the other two datasets, based on the 100 kV and 140 kV X-ray spectrum for dual energy CTP.

Coronary CTA: From The 120 kV set of gray-scale images was reconstructed by merging 50% of the Sn 140 kV spectrum and 50% of the 100 kV spectrum to optimize spatial and contrast resolution for assessment of coronary artery stenosis. Slice thickness and reconstruction increment were set at 0.75 mm and 0.4 mm, and medium-soft tissue convolution kernel (B26f) was used. CCTA were analyzed using a dedicated workstation (Aquarius workstation; TeraRecon Inc, San Mateo, Calif). By consensus reading among three experienced readers (two radiologists and one cardiologist), the presence of coronary stenosis was visually defined with lesions with 50% and more stenosis in diameter as significant with a combination of transverse sections and automatically generated curved MPR images of the target vessels. Coronary segments those were non-assessable because of extensive calcium and the presence of motion artifacts, were assumed to be having significant disease for the purpose of statistical analysis. When multiple lesions were present, the corresponding segment was classified by the worst lesion. Coronary arteries and lesions were segmented using a standard 15-segment model, and classified into 3 major coronary vessels: the left anterior descending artery (LAD), the left circumflex artery (LCX), and the right coronary artery (RCA). A ramus intermedius was classified to the LCX, if present.

**Figure 2:**
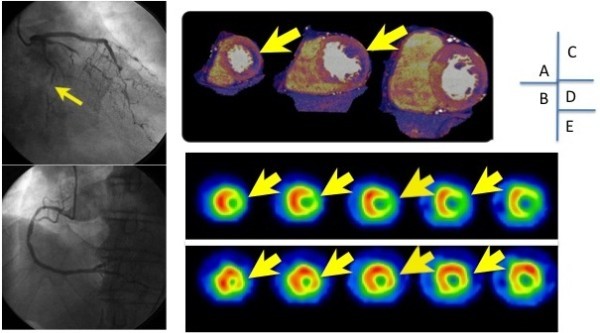
**Case (CAD = coronary artery disease, SPECT = single photon emission computed tomography).** Case was a patient with asymptomatic CAD. Severe stenosis is seen in the left circumflex (panel **A**), and RCA is normal (panel **B**). Stress dual-energy imaging shows ischemia in the lateral wall (panel **C**), which correlated with the lateral wall ischemia seen on SPECT (panels **D** and **E**).

Dual-energy CTP: Using two data sets, based on the 100 kV and 140 kV X-ray spectrum, the myocardial blood pool was analyzed by determining the iodine content within the myocardium on the basis of the unique X-ray absorption characteristics of this element at different kilovolt settings (Johnson et al. 2007; Flohr et al. 2006; Ruzsics et al. 2008). With dual-energy CT imaging technique, myocardial iodine distribution was calculated from the dual-energy data using commercially available software (Syngo Dual Energy, Siemens Healthcare, Forchheim, Germany). Color-coded iodine maps were carefully superimposed onto “virtual non-contrast (VNC)” reconstruction images, and consecutive series of the multiplanar reformats in cardiac short-axis view with 8 mm thickness were reconstructed. Cold colored (dark purple) myocardium in the color-coded CTP image was defined as positive dual-energy CTP by two independent radiologists, who were blind toward other diagnostic test results (Figure 2). The LV myocardial segments were based on a standard 17-segment model, and classified into 3 major coronary vessels (Cerqueira et al. 2002).

Combined assessment with CCTA and dual-energy CTP: when CCTA was evaluable in given coronary vessel, presence or absence of significant stenosis on CCTA defined positive or negative combined assessment of two tests. While when CCTA was not evaluable, positive or negative dual-energy CTP defined those of combined assessment.

### CAG

CAG was performed with 5-Fr catheters using standard techniques via the radial approach. At least minimum 8 projection images were obtained (5 views for the left coronary artery and 3 views for the right coronary artery). All CAG images were quantitatively evaluated using commercially available software (QCA-CMS system version 3.0, MEDIS, Leiden, The Netherlands) by two cardiologists. Coronary arteries, were classified into 3 major coronary vessels as same as CCTA. Significant coronary stenosis was defined with more than 75% luminal narrowing in diameter as standard reference.

### Statistical analysis

We compared the diagnostic accuracy for ischemic region due stenosis by ATP stress dual energy CT (only cardiac CTA imaging and combined coronary CTA and myocardial CT perfusion imaging) with invasive coronary angiography, agreement were calculated using Fisher’s exact probability test. All statistical analyses were performed using SPSS software (version 19; SPSS, Chicago, IL, USA). A probability value of less than 0.05 was considered statistically significant.

## Results

### Dual-energy CT

All 21 patients completed the ATP stress dual-energy CT protocol without significant side effects. However, 11 patients complained of transient flushing, and six patients complained of transient chest discomfort. None of the patients required aminophylline infusion to reverse the adverse effects. The mean heart rate was significantly higher in the ATP post-stress state (70.3 ± 11.5 beats/min) than in the ATP pre-stress state (62.1 ± 4.8 beats/min). The average radiation dose for those undergoing dual-energy CT was 7.7 ± 2.8 mSv (Definition 9.7 ± 0.7 mSv, Definition Flash 6.3 ± 3.0 mSv).

### Coronary CTA

Among the 21 CCTA examinations, 9 patients had at least one nonevaluable vessel due to severe calcification, and the remaining 12 patients were completely assessed on 3 major coronary vessels. The ratio of the evaluable vessels was 71.4% (45/63). CCTA depicted 12 stenotic vessels in 12 patients. Clinical prevalence of stenotic vessels was 47% (30/63) including 18 unevaluable vessels as clinical stenosis.

### Coronary CTA combined with CT perfusion

With Combined CCTA/CTP evaluation, ischemic territory was detected in 19 of 63 main coronary territories among the 21 patients. Of the 19 territories, 8 corresponded with the left anterior descending (LAD) artery, 6 corresponded with the left circumflex artery (LCX), and 5 corresponded with the right coronary artery (RCA).

### CAG

In conventional CAG, significant stenosis (≥ 75%) was detected in 24 of 63 main coronary vessels among 21 patients: 9 patients had one-vessel disease, six patients had two-vessel disease, and one patient had three-vessel disease. Of the 24 stenoses, 12 were in the LAD artery, five were in the LCX, and seven were in the RCA.

### Diagnostic accuracy of CCTA alone and CCTA combined with CTP (combined CCTA/CTP)

In comparison with CAG coronary stenosis per-vessel basis, agreement between CCTA alone and combined CCTA/CTP was 83% (52/63).

For detecting obstructive CAD, the sensitivity, specificity, PPV and NPV were 83.3% (20/24), 74.4% (29/39), 66.7% (20/30), and 87.8% (29/33) for CCTA alone, and 66.7% (16/24), 92.3% (36/39), 84.2% (16/19), and 81.8% (36/44) for combined CCTA/CTP, respectively (Table 1). The diagnostic accuracy of the two methods were 77.8% (49/63) and 82.5% (52/63).

**Table 1:** **Diagnostic accuracy and assessability for coronary CTA alone and combined CCTA and dual-energy CTP**

	Sensitivity	Specificity	PPV	NPV	Accuracy
CCTA alone	20/24	29/39	20/30	29/33	49/63
	83.3%, 62.6–95.2%	74.4%, 57.9–86.9%	66.7%, 47.2–82.7%	87.9%, 71.8–96.5%	77.80%
CCTA/CTP	16/24	36/39	16/19	36/44	52163
	66.7%, 447–84.3%	92.3%, 79.1–98.3%	84.2%, 60.4–96.4%	81.8%, 67.3–91.8%	82.50%

CCTA = coronary computed tomography angiography; CTP = computed tomography perfusion.

Data are presented as n/N (%, 95% confidence interval).

## Discussion

Diagnosis of a myocardial perfusion abnormality is an important step for the assessment of the extent and severity of myocardial ischemia and for risk stratification in patients with CAD. Clinically, myocardial perfusion is assessed using SPECT, contrast echocardiography, magnetic resonance imaging (MRI), invasive coronary catheter examination or positron emission tomography (PET) (Miyagawa et al. 1995; Nagel et al. 2003; Kaul et al. 1997). Recently, several reports of myocardial perfusion imaging using MSCT have stated that ECG-gated MSCT was able to depict ischemia in patients with CAD and that findings generated by this imaging modality correlated with those seen on SPECT and CAG. (Kurata et al. 2005; George et al. 2009; Kido et al. 2008; Shikata et al. 2010). The DSCT scanner has allowed broader application of dual-energy contrast-enhanced imaging, since the two orthogonally mounted detectors and tube arrays operate simultaneously and can be set to different tube potentials, enabling dual-energy CT acquisitions with minimal registration artifacts due to patient motion. Maps of iodinated contrast material content can be extracted without the need for complex image registration, which is invariably required with traditional single source CT. Recent several studies have shown that dual-energy CTP is promising for assessment of myocardial ischemia and infarction (Ruzsics et al. 2008, 2009; Ko et al. 2012; Kang et al. 2010; Bauer et al. 2010). In the present study, the iodine map could detect ischemia with greater clarity during ATP stress. Coronary artery stenosis doesn’t always involve myocardial ischemia, and identification of significant hemodynamically stenosis is not often straightforward, even if seen in CCTA.

In assessment of CTP imaging, optimization of scan timing is essential, because ischemic myocardial low attenuation is transient if the input contrast medium is interrupted. Our routine scan protocol of CCTA is set at 5 s after the predefined threshold, while present of stress dual-energy CT was set at 8 s after the threshold using longer contrast infusion (100 ml; 5 ml/s for 20 s) to scan time, taking our previous dynamic CTP studies and robust clinical use into account. As the result, we were able to assess coronary artery and myocardial perfusion, simultaneously with single image acquisition, even if coronary artery stenosis and the unevaluated legions due to calcification were seen in CCTA.

DSCT was performed with parameters of 140 and 80 kV, which are often used because dual-energy CT requires a large difference in the tube voltage energy. However, iodine maps with parameters of 140 and 80 kV have low signal-to-noise ratios, resulting in decreased diagnostic accuracy, particularly for myocardial ischemia. Use of Sn 140 and 100 kV can double the radiation dose (mSv) when compared with the use of 140 and 80 kV. Consequently, use of Sn 140 and 100 kV decreases imaging noise and increases the reliability of iodine maps. Schenzle et al. recently reported that there was no difference in the effective radiation dose measured with the thermoluminescent detectors between dual-energy mode at Sn 140 and 100 kV and the standard 120 kV scans. Further, dual-energy CT is feasible without an additional radiation dose (Schenzle et al. 2010).

### Limitations

The present study had several limitations. First, the number of patients was relatively small. Second, because the renewal of the DSCT, we used different DSCT (Definition and Definition Flash). However, there was no indication that using the different DSCT affected the results. Third, CTP should be validated with other modalities, for instance, MRI, SPECT and PET. Forth, myocardial ischemia should be assessed with stress image and rest image. But rest scanning was not performed in this study in order to limit the radiation dose and the amount of contrast medium. In this regard, dynamic perfusion MRI may be more beneficial than MSCT, while the availability of MRI is limited, because of time-consuming, technical difficulty and lower patient throughput. Lastly, the integration of CCTA and dual-energy CTP with single image will allow for more precise and effective diagnosis.

## Conclusion

Dual-energy CT may be a useful modality for perfusion assessment and correlated well with the severity of stenosis on CAG. This technique may even be of use in cases of severe calcification in the coronary artery wall.
